# Female-specific genomic regions and molecular sex identification of the clearhead icefish (*Protosalanx hyalocranius*)

**DOI:** 10.1186/s12864-021-07830-9

**Published:** 2021-07-02

**Authors:** Teng-Fei Xing, Yu-Long Li, Jin-Xian Liu

**Affiliations:** 1 CAS Key Laboratory of Marine Ecology and Environmental Sciences, Institute of Oceanology, Chinese Academy of Sciences, 266071 Qingdao, China; 2 Laboratory for Marine Ecology and Environmental Science, Qingdao National Laboratory for Marine Science and Technology, 266237 Qingdao, China; 3 Center for Ocean Mega-Science, Chinese Academy of Sciences, 266071 Qingdao, China; 4 University of Chinese Academy of Sciences, Beijing, China

**Keywords:** Clearhead icefish, Female-specific marker, ZZ/ZW sex determination system, Genetic sex identification

## Abstract

**Background:**

The clearhead icefish, *Protosalanx hyalocranius*, is an economically important fishery species in China. Since 1980s, *P. hyalocranius* was widely introduced into lakes and reservoirs of northern China for aquaculture. However, the lack of a rapid and cost-effective sex identification method based on sex specific genetic markers has hindered study on sex determination mechanisms and breeding applications.

**Results:**

Female-specific genomic regions were discovered by comparing whole genome re-sequencing data of both males and females. Two female-specific genomic regions larger than 50 bp were identified, and one (598 bp) contained a putative FOXI gene, which was paralogous to another FOXI gene with sex-associated SNPs. The two FOXI sequences displayed significant length difference with nine deletions of total length of 230 bp. This deletion-type structural variation could be easily and efficiently detected by traditional PCR and agarose gel electrophoresis with one 569 bp band for males and two bands (569 and 339 bp) for females, which were validated in 50 females and 40 males with known phenotypic sexes.

**Conclusions:**

The results provided structural genomic evidence for the ZZ/ZW sex determination system in *P. hyalocranius* discovered in our previous study with association analysis of SNPs. Moreover, the female-specific markers and rapid and cost-effective PCR-based genetic sex identification method should have applications in further studies of sex determination mechanism for this species.

**Supplementary Information:**

The online version contains supplementary material available at 10.1186/s12864-021-07830-9.

## Background

Sex determination is a fundamental question in life science, and has been considered as the queen of all problems in evolutionary biology [1]. Due to its great implications in both theory and practice, the mechanism of sex determination in fish has aroused so much biologist’s attention [2]. As primitive vertebrates, teleost fishes possess complicated and diverse sex determination systems [3, 4]. Different sex determination systems have been reported even among closely related fish species in the same genus [5, 6]. Therefore, researches of sex determination in fish will provide insight into the evolution of sex determination in vertebrates [7]. Moreover, many farmed fishes display sexual dimorphisms between males and females, such as body size and growth rate [4, 8, 9]. For example, males grow faster than females in southern bluefin tuna [10], channel catfish [11] and Nile tilapia [12]; while in some other fishes, such as half-smooth tongue sole [13], sea bass [14] and rainbow trout [15], females grow faster and larger than males. However, many fish species can not be sexually distinguished until adult or sexual maturity stage (e.g. ayu [16] and large yellow croaker [17]), which greatly hindered identification of sex in fishes at early life stages. Hence, revealing the mechanism of sex determination and developing a rapid and cost-effective sex identification method are critical to the understanding of the reproductive biology of artificially cultured species and genetic factors involved in sex differences [18, 19], and for the guiding of artificial breeding programs.

Three approaches were commonly adopted in studies of the sex determination system, i.e., cytogenetic approaches, breeding experiments, and identification of sex-specific molecular markers [20]. Cytogenetics approaches may be problematic in fishes while most species lack visually heteromorphic sex chromosomes [2]. Breeding experiments are also limited because it mainly focuses on species with successful breeding techniques. Therefore, identification of sex-linked or sex-specific markers have been considered as a powerful approach to study the genetic basis of sex determination in the widest variety of species [21, 22].

Sex-specific markers exist on the heterogametic sex chromosome, the Y in species with male heterogamety or the W in species with female heterogamety, which could be used to determine whether a species has genetic sex determination (GSD) with either male or female heterogamety [21]. Generally, the presence of a male-specific marker indicates an XX/XY system, while the presence of a female-specific marker indicates a ZZ/ZW system [23]. Over the past few decades, various molecular methods have been developed to explore sex-specific markers in aquaculture fishes including random amplified polymorphic DNA (RAPD) in rainbow trout (*Oncorhynchus mykiss*) [24], African catfish (*Clarias gariepinus*) [25], Nile tilapia (*Oreochromis niloticus*) [12], and turbot (*Scophthalmus maximus*) [26]; amplified fragment length polymorphism (AFLP) in rainbow trout (*O. mykiss*) [15], half-smooth tongue sole (*Cynoglossus semilaevis*) [13], gibel carp (*Carassius auratus gibelio*) [27], and bagrid catfish (*Pseudobagrus ussuriensis*) [28]; and microsatellite markers in half-smooth tongue sole (*C. semilaevis*) [19] and rock bream (*Oplegnathus fasciatus*) [29]. However, identification of sex-specific markers using these traditional molecular techniques is usually inefficient and expensive. Recently, the next-generation sequencing (NGS) approach has greatly improved the efficiency of research on sex-determination, making the identification of sex-specific markers much cost-efficient from a whole genome scale. For example, sex-specific markers were identified in some species using restriction site associated DNA sequencing (RAD-Seq) [30–33]. However, most of these studies discovered sex-specific markers through construction of linkage maps from test crosses. Unfortunately, for fishes that were not easily bred in captivity or with long generation times, sequencing parents and offspring may be not feasible. Therefore, whole genome sequencing (WGS) and sex-association analysis could be more appropriate to detect sex-specific markers for these fishes [3, 34].

The clearhead icefish, *Protosalanx hyalocranius*, a diadromous fish belonging to the family of Salangidae, mainly inhabits in coastal areas and adjacent freshwaters of Korea and China [35, 36]. Members of Salangidae inhabit fresh, brackish and coastal waters of the Far East, from Vietnam to Sakhalin, and are known by many colloquial names (e.g., icefishes, salangids and noodlefishes) [37, 38]. *P. hyalocranius* is an important commercial fishery species in China with a wide geographical distribution [39]. Wild populations of *P. hyalocranius* have markedly declined in recent years due to over-exploitation, hydroprojects and water pollution [40]. *P. hyalocranius* has also been widely introduced into lakes and reservoirs of northern China for aquaculture [39]. Artificial breeding techniques have been established and seedlings have been released to lakes and reservoirs to improve aquaculture yield [41]. Like other Salangid fishes, *P. hyalocranius* has annual life cycle and dies after spawning. Salangid fishes are special among teleosts because of sexual dimorphism at maturation, which includes: (1) single row of scales at the base of anal fin, which was not present in female; (2) anal fin height of males is greater than females. (3) first ray of pectoral fin longer and pointed in males; (4) body height at anus greater in males than females [42]. However, no phenotypic differences exist between males and females before sexual maturation. Additionally, previous cytogenetic studies of *P. hyalocranius* suggest that there are no visually heteromorphic chromosomes [43]. Recently, by analyzing genome-wide sex-associated SNPs, a ZZ/ZW sex determination system is identified for *P. hyalocranius* [44]. However, a rapid and cost-effective genetic sex identification method is still lacking, which hinders sex identification of immature individuals in ecological studies and aquaculture breeding of this species. Thus, female-specific genetic markers and rapid and cost-effective method for genetic sex identification at early life stages are needed for *P. hyalocranius*.

In the present study, first we aim to detect female-specific genomic regions by comparing whole genome re-sequencing data from both males and females of *P. hyalocranius*. Then a rapid and cost-effective molecular method was developed for genetic sex identification of *P. hyalocranius*. Further, the developed molecular sex identification approach was used to verify the ZZ/ZW sex determination system in *P. hyalocranius* that was identified in our previous study. These findings will provide insights into the mechanism of sex determination and evolution of sex chromosome in Salangid fishes.

## Results

### Identification of female-specific genome regions in *P. hyalocranius*

**Fig. 1:**
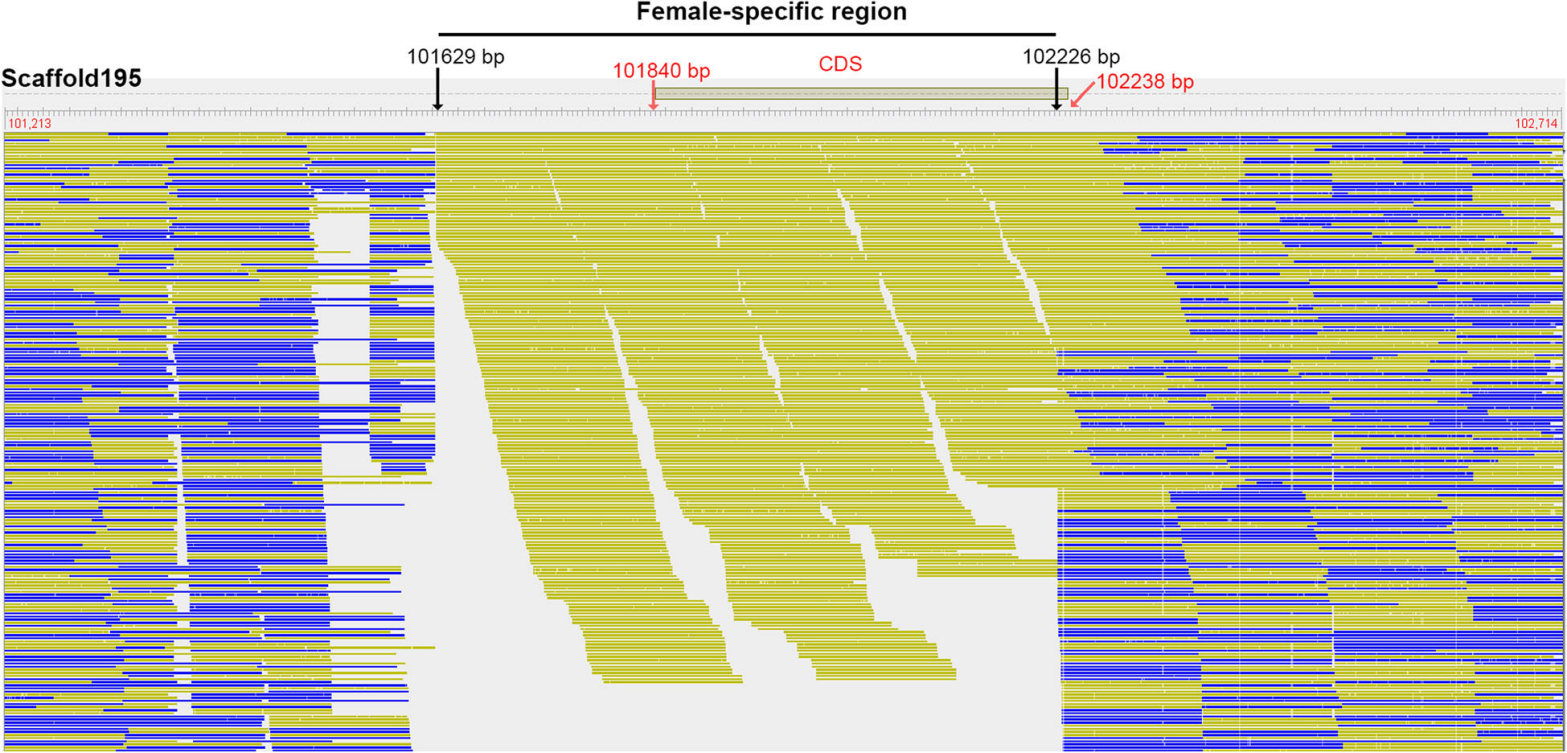
Sequencing reads alignments showing the female-specific genome region on scaffold195. Lines in blue represent reads from males, while lines in yellow represent reads from females. The female-specific region spans 598 bp, ranging from 101,629 to 102,226 bp. The yellow box on the top represents the CDS region, ranging from 101,840 bp to 102,238 bp

Mapping results indicated that the sequenced reads covered most of the reference genome, with breadth of coverage ranged from 72.71 to 74.58 % among individuals, and a mean depth of 12.88 in females and 14.43 in males respectively. After comparing the reads depth between females and males, two genomic regions that were unique to females with length larger than 50 bp were detected (Additional file 1 Table S1). The two regions were both located on scaffold195 (923,714 bp) and with length of 598 and 134 bp respectively. The physical position of the larger female specific genomic region ranged from 101,629 bp to 102,226 bp on scaffold195 (Fig. 1). The average depth of coverage in this region was 6.60 in females (approximately half of the overall mean depth for the diploid *P. hyalocranius*), suggesting it was a haploid copy specific to females. However, the corresponding depth of this region was zero for males, which further supported the ZZ/ZW sex determination system in *P. hyalocranius*.

After examining the gene predictions file, we found a gene ID = LS_GLEAN_10009963 present in the 598-bp female specific region, which located from 101,840 bp to 102,238 bp on scaffold195. This gene contained only one exon and encoded a putative protein of 132 amino acids, which was named as the female-specific gene. BLASTp searching showed that the female-specific gene was homologous to a FOXI gene (FOXI2, ID = LS_GLEAN_10021007) shared by both sexes, that had two exons with 372 amino acids and contained sex-associated SNPs on scaffold4 [44]. Furthermore, a total of five FOXI genes were identified in the genome of *P. hyalocranius* and phylogenetic analysis suggested that the female-specific gene (LS_GLEAN_10009963) was closely related to the FOXI2 gene (LS_GLEAN_10021007) (Additional file 1 Fig. S1). Alignment of amino acid sequences suggested that the female-specific gene and its closely related FOXI gene shared ~ 75 % similarity (Additional file 1 Fig. S2).

**Fig. 2:**
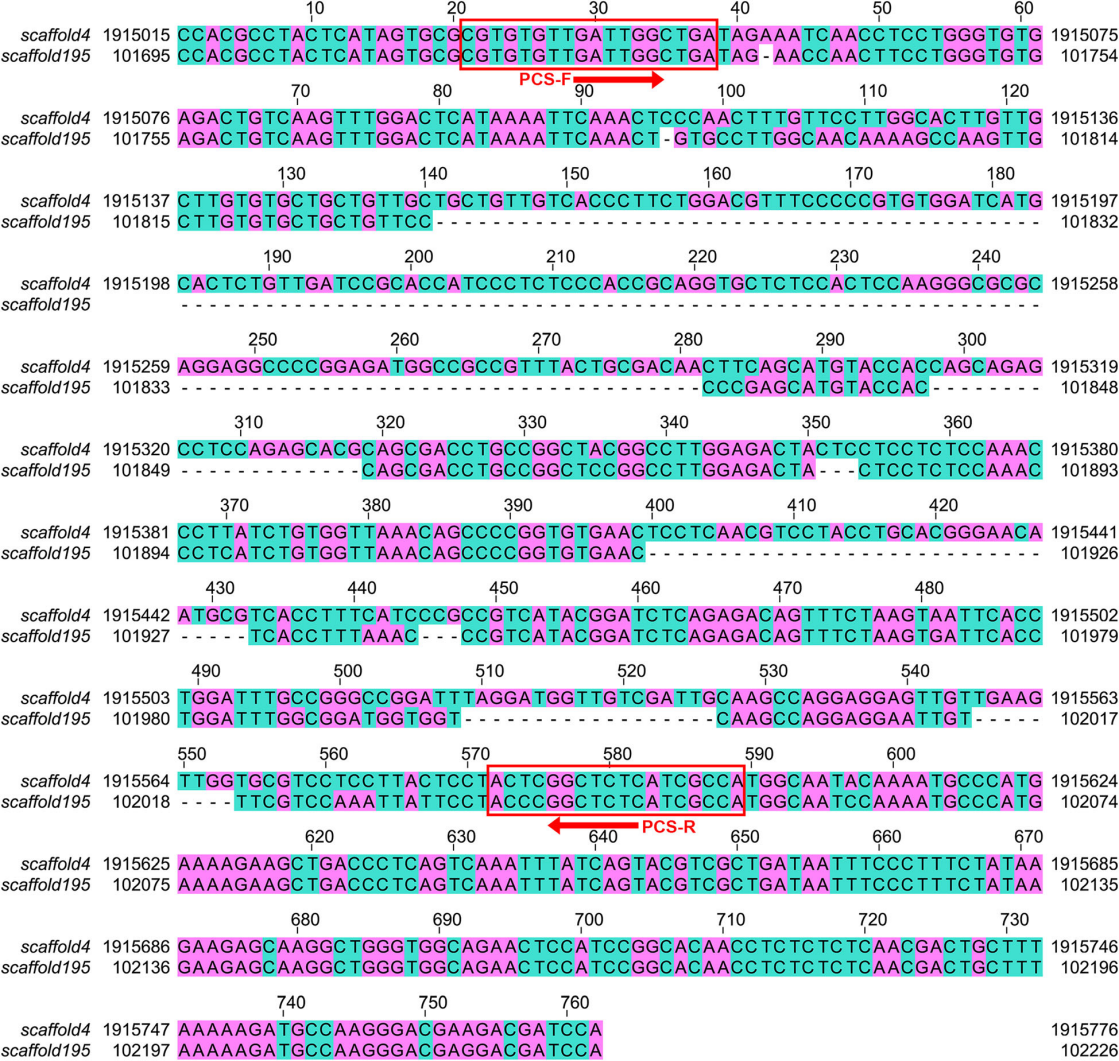
Alignments of the longer sequence (female and male shared) on scaffold4 and shorter sequence (female specific) on scaffold195. The primers PCS-F and PCS-R are displayed in the red frame. Sequence positions: scaffold4 from 1,915,015 bp to 1,915,776 bp, scaffold195 from 101,695 bp to 102,226 bp

DNA sequence alignments revealed that there were nine gaps (total size 230 bp) between the haploid female-specific region on scaffold195 and the paralogous diploid sequences on scaffold4 (Fig. 2 and Additional file 5), which made them suitable for rapid and cost-effective genetic sex identification.

### Primer design and verification

**Fig. 3:**
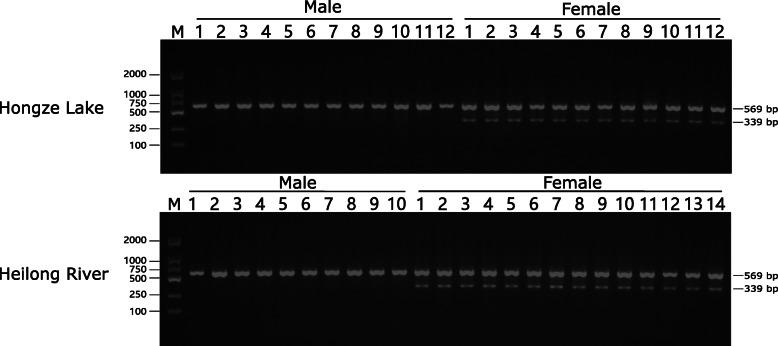
PCR amplification results using PCS primer pair in 48 individuals (12 males and 12 females from Hongze Lake, 10 males and 14 females from Heilong River). The 569 bp long band (shared by males and females) and the shorter 339 bp female specific band are indicated respectively. The DL 2000 DNA marker (Sangon Biotech Co., Ltd) is shown in the left. This picture is a cropped gel and the uncropped gel was presented in Additional file 1 Fig. S3

A set of PCR primers was designed according to the consensus flanking sequence of the haploid female-specific region and its paralogous diploid sequences (Fig. 2). The aligned sequences were 532 bp for the female-specific region on scaffold195 and 762 bp for the paralogous region on scaffold4, respectively (Fig. 2). The primers, which located in the highly conserved flanking region of the two sequences, resulted in two target sequences of different lengths (569 and 339 bp) in females and one target sequence of 569 bp in males. PCR was successful in the preliminary test with four female individuals and four male individuals, which clearly showed two bands in females and one band in males. Sanger sequencing of PCR products from one female and one male verified the nature of the target sequences (Additional file 2 , 3, 4). Further test based on 90 individuals with known phenotypic sex (30 males and 30 females from Hongze Lake, 10 males and 20 females from Heilong River) consistently displayed two bands in females and one bands in males (Additional file 1 Fig. S3). Yield of PCR product of the short band unique to female was apparently less than that of the long band, which confirmed that the short female-specific sequence was haploid and the homologous sequence was diploid in both sexes (Fig. 3). Thus, this primer should be an ideal tool/resource for rapid and cost-effective genetic sex identification in *P. hyalocranius.*

## Discussion

More than 32,000 species of fish inhabit a wide range of aquatic habitats worldwide, which provide a rich source of material for studying vertebrate sex determination [2, 45]. Icefishes have been commercially exploited for a long history in China. To the best of our knowledge, few studies concerning sex determination system of Salangidae were conducted during the past few decades. Recently, the genome of *P. hyalocranius* has been sequenced, which can enhance our ability to investigate the sex determination system and development of sex-specific markers. In this study, two female-specific genomic regions were discovered based on NGS data, and a PCR-based method was developed for the rapid and cost-effective identification of sex in *P. hyalocranius*, which should have applications in further research on the molecular mechanism of sex determination and breeding for this species.

Compared with traditional sex identification methods, the detecting strategy developed in our study had following advantages. Firstly, our strategy was time-saving and accurate. For instance, RAPD technique requires strict standardization of PCR conditions because different concentrations of DNA polymerases, DNA template and primer ratios or annealing temperatures can lead to differences in amplification results, resulting in low repeatability in fingerprints [46]. Secondly, the NGS-based method is highly effective in detection of sex-specific markers due to its wide breadth of genome coverage compared with AFLP or RAD-seq based methods. Because of the non-random distribution of endonucleases cut sites in the genome, substantial sex-specific regions may escape from AFLP or RAD-seq scanning [47, 48]. The genomic DNA was randomly sheared and then sequenced by NGS and could cover most of the genome [22]. Even with the whole genome re-sequencing data, only two female specific fragments larger than 50 bp were detected in our study, which suggested that the NGS-based approach is particularly effective in detecting sex-specific markers for species with primitive sex chromosomes without apparent divergence. Thirdly, unlike SNP based method, the haploid female-specific region discovered is paralogous to another gene in the genome with large length difference, which makes it ideal to identify both sexes simultaneously and accurately. Generally, following the detection of sex-specific markers through SSRs or NGS method, identification of sex could be achieved by simple PCR-based approach. However, genetic sex identification based on sex-linked SNPs needs to design specific primer containing mismatched nucleotides in a single sex. Positive PCR amplification band will display in females or males but absent in another sex. In this scenario, we can’t distinguish whether the negative PCR amplification is due to primer mismatch or just false negatives. Therefore, in order to identify the genetic sex of an individual efficiently and correctly, simultaneous PCR amplification of a marker shared by both sexes and a sex-specific marker should be highly effective. Genetic sex identification through PCR and gel electrophoresis more than one time is time-consuming and step-cumbersome. Besides, genetic sex identification methods were also developed based on sex-specific deletions in homologous genomic regions [17, 22, 49]. In these studies, PCR amplification results in two bands in one sex and a single band in the other, which is the same as in the present study [3, 34]. However, if the size of the sex-specific deletion was small, the two close bands could not be easily distinguished from each other in low resolution gel electrophoresis. For *P. hyalocranius*, the large size difference between the female-specific sequence and its paralogous diploid counterpart could be easily distinguished from each other via routine agarose gel electrophoresis. The primer set we developed can simultaneously amplify both the female-specific genomic region and the paralogous sequences shared by both sexes, and effectively identify males and females using a single PCR reaction. Our PCR-based genetic sex identification approach is simple and convenient in practice, which would be a powerful and effective tool to understand the reproductive biology of *P. hyalocranius*. This approach will also have important applications in detecting the genetic factors involved in sex differences and uncovering the evolution of sex chromosome.

Heterogametic sex chromosomes are usually present in mammals and birds. However, distinguishable sex chromosomes have only been observed in less than 1 % of teleost (~ 270 species) [50, 51] such as half-smooth tongue sole (*C. semilaevis*) [52] and nine-spined stickleback (*Pungitius pungitius*) [53]. A previous cytogenetic study demonstrated that *P. hyalocranius* possessed 28 pairs of chromosomes and no heterogametic sex chromosomes were detected [43], which hindered study of sex chromosome and sex determination mechanism using traditional cytogenetic techniques. Besides, the secondary sexual characters appear only in males when they are getting sexually mature, which indicates that the males and females could not be distinguished morphologically in most stages of the life cycle. Hence, the sex-specific marker developed in our study is crucial for identifying the genetic sex of *P. hyalocranius* at early stage of life cycle for ecological studies and molecular breeding applications.

Results of the present study have important meanings to both fundamental and applied research. The lack of sex-specific DNA markers has hindered the investigation of sex determination mechanisms for this species. To date, sex-specific markers have been developed in many fish, while sex determining genes were only identified in a few species. For instance, *dmrt1*, which is the male-determining gene in birds, showed convergent evolution of features and are compatible with a similar function in tongue sole (*C. semilaevis*) [52]. Knocked out *dmrt1* in *C. semilaevis* confirmed its important role in sex determination [54]. The present study validated a ZZ/ZW determination mechanism in *P. hyalocranius*, which was consistent with our previous study [44]. However, the master sex-determining gene has not been identified yet. Studying the differential expression of sex-related or sex-determining genes in females and males is prerequisite for elucidating the molecular mechanisms of sex determination and development. Since female and male of *P. hyalocranius* are indistinguishable during the embryo, larvae and juvenile stages, a sex-specific marker is required to identify their genetic sex. Thus, the convenient PCR-based sex identification method will promote the study of molecular mechanisms for sex determining in *P. hyalocranius*.

## Conclusions

In the present study, female-specific genomic regions for *P. hyalocranius* were identified based on NGS data. One 598 bp female-specific haploid sequence containing a putative FOXI gene was paralogous to a FOXI2 gene with sex-associated SNPs detected in our previous study. A total length difference of 230 bp between the female-specific sequence and the paralogous gene were detected. A PCR-based method was developed for rapid and cost-efficient genetic sex identification in this species. The sex-specific markers and PCR-based method should have applications in elucidating the molecular mechanism of sex determination and breeding biotechnologies in this species.

## Methods

### Sample collection, DNA extraction and raw reads processing

Whole-genome resequencing data from our previous study [44] were reused for analysis, which consisted of 20 males and 20 females collected from Hongze Lake (N 33°16´, E 118°44´) on December 2018. Details about samples collection, phenotypic sex determination, DNA extraction, library preparation, sequencing and raw reads processing were described in Li et al. [44]. In addition to the 40 individuals used for whole-genome resequencing, genomic DNA of another 50 samples with known phenotypic sex (10 males and 10 females collected from Hongze Lake on December 2018, 10 males and 20 females collected from Heilong River on January 2019) were extracted for PCR verification after sex-specific markers were developed. The average body size of these fishes was approximately 15 cm.

### Identification of female-specific genomic regions

Genome-wide SNPs analysis indicated that the individual used for the draft reference genome sequencing and assembly by Liu et al. [55] was a heterogametic female (ZW). Thus, this reference genome was suitable for the identification of female-specific genomic regions. In order to identify female-specific genomic regions, for a species with ZZ/ZW sex determination system like *P. hyalocranius*, we used the following method to extract the genome regions that were unique to female. First, reads from different individuals of both female and male were aligned to the reference genome using BWA mem v0.7.17 [56]. Second, coverage of depth for each individual was extracted using SAMtools v1.10 [57], and their significances between two sexes were accessed using Welch’s t-test. Only genome regions with overall depth of coverage ≥ 5 in at least one sex group were used. Third, genome regions showing significant differences (*P*-value < 1e-4) of depth of coverage between two sexes were identified, and regions that were present only in females were extracted and used for downstream primers design and annotations. The alignment files for the target regions showing sex specificity were manually examined using Tablet v1.21.02.08 [58]. Gene predictions located in the sex-specific regions were extracted from the annotation file provided by Liu et al. [55], and the protein sequences were queried using the NCBI non-redundant protein sequences (nr) database by BLASTp online (NCBI) to retrieve the putative functions.

### Phylogenetic analysis of the female-specific gene and paralogous genes

Since the female-specific genomic regions contained a putative FOXI gene, we then retrieved the protein sequences of all FOXI genes in the *P. hyalocranius* genome annotation file using local BLASTp as implemented in BLAST + v2.11.0 [59]. The protein sequences were then aligned to each other by MAFFT v7.471 [60], and a maximum likelihood phylogenetic tree was constructed by IQTREE2 v2.1.3 [61] using 1000 ultrafast bootstraps. The best substitution model was chosen based on BIC score using ModelFinder as implemented in IQTREE2 v2.1.3.

### Development and test of a sex-specific marker

Female specific regions were aligned to the reference genome of *P. hyalocranius* using local BLASTn as implemented in BLAST + v2.11.0 [58], and homologous sequences were aligned by MAFFT v7.471[59]. A 598 bp female specific genomic region was found paralogous to another genomic region shared by both females and males, but with large length difference, suggesting an ideal system for rapid genetic sex identification by traditional PCR and gel electrophoresis. A primer pair (PCS-F: CGTGTGTTGATTGGCTGA; PCS-R: TGGCGATGAGAGCCGAGT) was designed in the consensus flanking sequence of the female-specific region and its homologous genomic region using the Primer Premier 5.0 software (http://www.premierbiosoft.com/). Genomic DNA from four female individuals and four male individuals were first used as templates to test the validity of this marker for sexing. Each PCR reaction was carried out in a final volume of 10 µl containing 1 µl DNA template, 5 µl 2xTaqMasterMix (Dongsheng Biotech Co., China), 0.25 µl each primer (10 µM) and 3.5 µl ddH_2_O. PCR were carried out using the following cycling conditions: pre-denaturation at 95 °C for 5 min; 35 cycles of denaturation at 95 °C for 20 s, annealing at 56 °C for 30 s and extension at 72 °C for 30 s; a final extension of 5 min at 72 °C. PCR products were visualized using 1 % agarose gel.

### Accuracy of genetic sex identification base on PCR amplification

In order to test the validity of the sex identification method, 90 matured clearhead icefishes with known phenotypic sexes were used to test the accuracy of this method. PCR were carried out using the same conditions as mentioned above. To verify the amplified sequences, PCR products of one female and one male were sent to Sangon Biotech Co., Ltd for Sanger sequencing.

## Supplementary Information

**Additional file 1.** Supplementary table and figures.

**Additional file 2. **The original sequence file amplified by PCS-F and PCS-R in male. 

**Additional file 3. **Original sequence file of the shared sequence in female amplified by PCS-F and PCS-R.

**Additional file 4. **Original sequence file of the specific sequence in female amplified by PCS-F and PCS-R.

**Additional file 5. **Alignment file of the female-specific sequence and its paralogous sequence shared by both sexes.

## Data Availability

Sequencing data were deposited in the National Center for Biotechnology Information (NCBI) Sequence Read Archive under BioProject Accession Number PRJNA545572. Other data generated or analyzed during this study are included in the main paper and supplementary information files.

## Abbreviations

GSD: Genetic sex determination
RAPD: Random amplified polymorphic DNA
AFLP: Amplified fragment length polymorphism
NGS: Next-generation sequencing
RAD-seq: Restriction-site associated DNA sequencing
WGS: Whole genome sequencing
SNPs: Single nucleotide polymorphisms
PCR: Polymerase chain reaction
SSR: Simple sequence repeat
