# Supplementary material for: Female-specific genomic regions and molecular sex identification of the clearhead icefish (Protosalanx hyalocranius)
Source: BMC Genomics. 2021 Jul 2;22:495. doi: 10.1186/s12864-021-07830-9 (PMC8254354; doi:10.1186/s12864-021-07830-9)

Table S1 Summary of the female-specific genome regions.

| Scaffold name | Start | End | Length | Gene* | Annotation |
| --- | --- | --- | --- | --- | --- |
| scaffold195 | 101,629 | 102,226 | 598 | LS_GLEAN_10009963 | forkhead box protein I2/I3 like |
| scaffold195 | 103,651 | 103,784 | 134 | N/A | N/A |

*The id of gene located in this region. N/A: not available.

Fig. S1 The maximum likelihood tree constructed based on FOXI genes in *Protosalanx hyalocranius*. Values on the branch represent the ultrafast bootstrap support values (%), label marked by green box represents the female-specific gene found in this study.


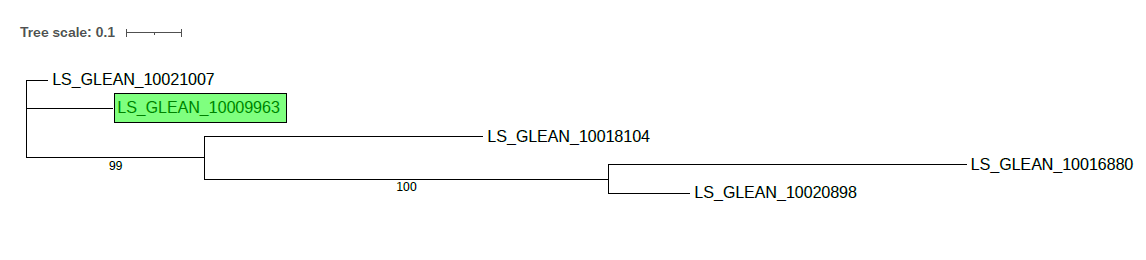


Fig. S2 Protein sequence alignments for the female-specific gene (LS_GLEAN_10009963) and the FOXI2 gene (LS_GLEAN_10021007) identified in our previous study.


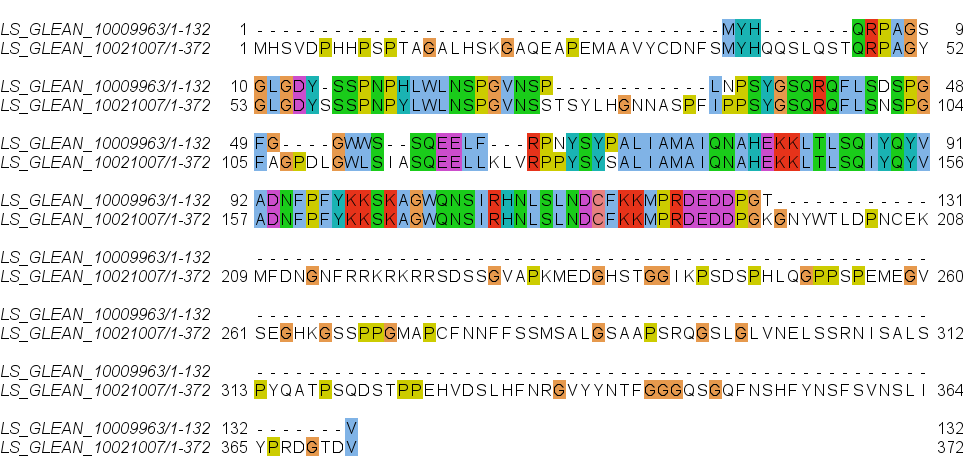


Fig. S3 The uncropped gel of PCR amplification results using PCS primer pair in 90 individuals. Individuals corresponding: (from left to right and top to bottom): 12 males and 12 females from Hongze Lake, 10 males and 14 females from Heilong River, 18 males and 18 females from Hongze Lake, and 6 females from Heilong River.


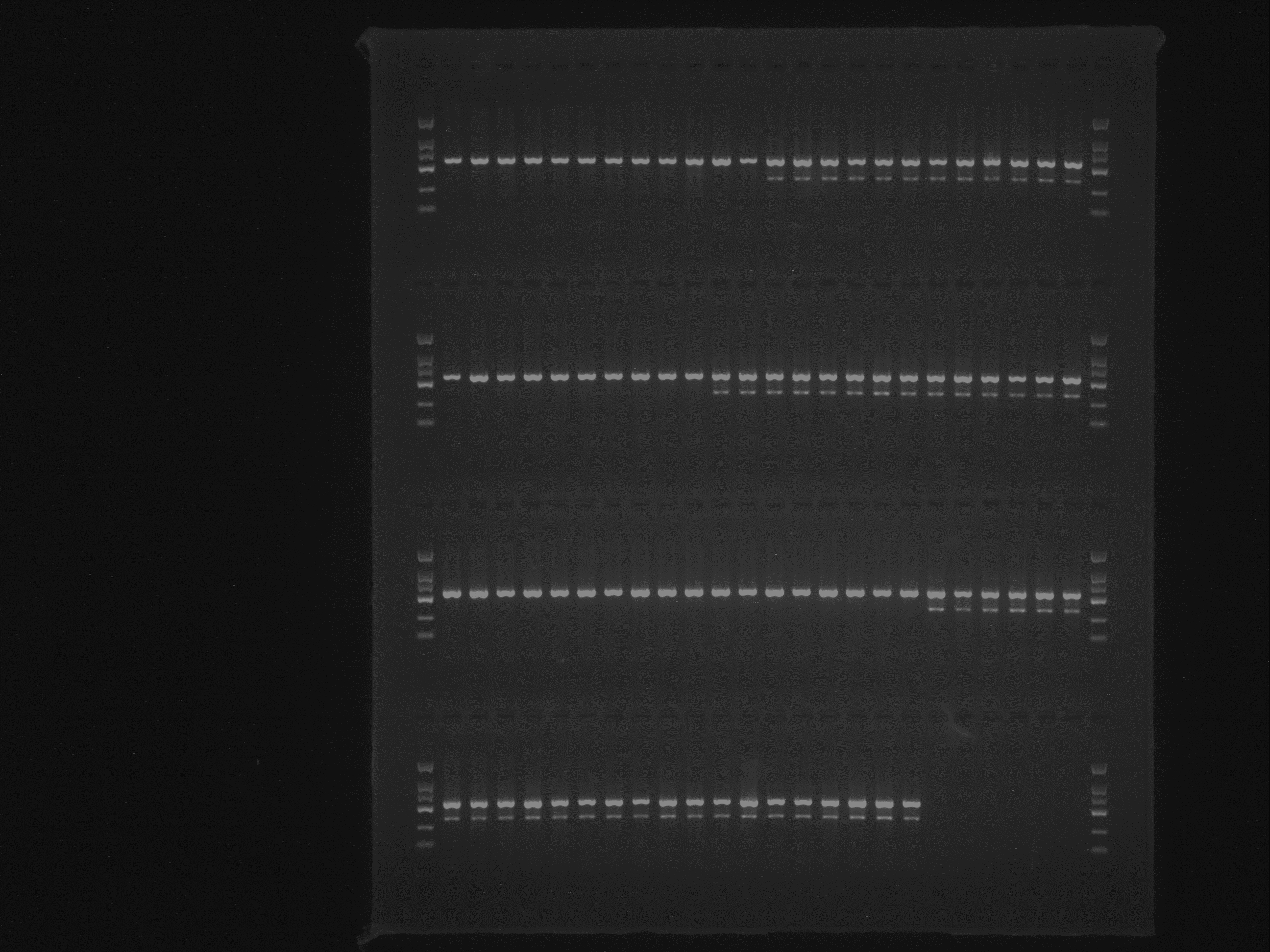

Supplement: Supplementary file 1 — Additional file 1. Supplementary table and figures. [file 12864_2021_7830_MOESM1_ESM.docx]
